# Influence of supplemental choline on milk yield, fatty acid profile, and weight changes in postpartum ewes and their offspring

**DOI:** 10.14202/vetworld.2024.1265-1270

**Published:** 2024-06-14

**Authors:** María M. Crosby-Galvan, German D. Mendoza, Pedro A. Hernández-García, José Antonio Martínez-García, Anayeli Vázquez-Valladolid, Rubén Oswaldo Cifuentes-López, Héctor A. Lee-Rangel

**Affiliations:** 1 Livestock Center, Postgraduate College, México State, México; 2 Agriculture and Animal Science Department, Xochimilco Campus, Metropolitan Autonomous University, Mexico City, México; 3 Mexico State Autonomous University, México State, México; 4 Agronomy and Veterinary Faculty, Bioscience Centre, San Luis Potosí Autonomous University, México

**Keywords:** birth, milk production, milk quality, thiocholine

## Abstract

**Background and Aim:**

The most intensive nutritional requirements occur during milk production’s peak. Ewe milk contains more protein and fat than cow milk. The nutritional factors significantly determine the composition. The liver undergoes high stress during lactation but is relieved by essential nutrients. Choline acts metabolically as a lipotrope. This compound functions in cell structure construction, maintenance, and acetylcholine synthesis. The animal nutrition industry provides choline from various sources, such as synthetic and natural kinds. This study evaluated the influence of two distinct choline sources on dairy ewes’ peripartum and postpartum milk production, composition, and offspring growth.

**Materials and Methods:**

Twenty-four Rambouillet ewes, each weighing around 63.7 ± 1.7 kg, aged three with two previous births, spent 30-day pre-partum and post-partum in individual pens (2 × 2 m). They were given different experimental treatments 30 days before and after birth according to a randomized design; no choline (a), 4 g/day rumen-protected choline (RPC) (b), or 4 g/day thiocholine (c). Milk samples for milk composition and long-chain fatty acid (FA) analysis were taken every 30 days during milk collection.

**Results:**

Significant differences (p < 0.05) in ewe body weight, lamb birth weight, and 30-day-old lamb body weight were observed at lambing and on day 30 of lactation due to choline treatment. Milk yield was significantly higher (1.57 kg/day) compared to the control (1.02 kg/day) and RPC (1.39 kg/day), due to the herbal choline source. There was no significant difference in the milk’s protein, lactose, fat, non-fat solids, and total milk solids content between the treatments. Herbal choline lowers (p < 0.05) the concentrations of caproic, caprylic, capric, lauric, and myristic acids while boosting (p < 0.05) those of oleic and cis-11-eicosenoic acid, the changes influencing long-chain FA levels (p < 0.05).

**Conclusion:**

Providing choline from both sources to ewes enhanced milk production and body weight at lambing and on 30-day post-lambing. The herbal choline supplement altered short-chain milk FAs, while representative concentration pathways affected medium-chain ones.

## Introduction

In late gestation, sheep and goats experience heightened fetal placental demand and reduced rumen capacity, leading to poor nutrition [1, 2]. Dairy ewes demand greater nutrient intake from late gestation to peak milk production. During the peripartum period, endogenous antioxidant levels are reduced [3]. The liver’s detoxification function can be overwhelmed by this condition. The offspring may undergo physiological and metabolic changes due to these factors [4]. Choline, a methyl donor in the diet, affects epigenetic regulation of gene expression by influencing DNA and histone methylation [5].

Choline contributes to three primary metabolic functions: Synthesizing acetylcholine, donating methyl groups through betaine, and producing phosphatidylcholine [6]. Betaine, an oxidized form of choline, acts as a methyl donor in the methionine cycle [7] and serves as the essential methyl donor for DNA, RNA, and protein methylation [8]. Methylated folate contributes to remethylating homocysteine into methionine by supplying methyl groups. Thus, choline supplementation contributes to the supply of methyl groups for methylation in the body [9].

The insufficient choline supply in growing lambs and high-yielding lactating animals’ diets has been shown by rumen-protected choline (RPC) supplementation studies, despite the undefined requirement for choline in sheep [10, 11]. Compared to the control group, about 16.7% faster suckling lamb growth for lactating ewes was achieved with rumen-protected methionine, choline, and betaine supplementation [12]. The dose of commercial RPC is established based on its choline content and bypass fraction. About 63%–98% of ruminal protection was indicated by *in vitro* ruminal incubations [13].

This plant source provides phosphatidylcholine [4]. The phosphatidylcholine transfer protein facilitates phosphatidylcholine transport between cellular membranes [6]. According to past research by NRC [10], and Martínez-García [14], a herbal product has been proven to replace RPC as a choline source in lambs. According to Martínez-García *et al*. [14], using an herbal choline source produces similar gains in average daily weight, meat appearance, and plasmatic metabolites for finishing lambs as compared to ruminant diet supplemented with RPC chloride [11, 13]. The majority of choline in herbal sources exists as lipid-bound total conjugates, thereby hindering ruminal degradation [4, 10, 14].

According to four experiments with dairy goats, a response of 200 g more milk/day occurred with RPC supplementation [15]. Therefore, we conjecture that dairy ewes may also exhibit a comparable response. Supplementing representative concentration pathways (RCP) may aid in the prevention of metabolic diseases like toxemia in dairy cows due to its effects on energy balance, feed intake, and hepatic lipid metabolism [16], and thiocholine supplementation has been shown to improve liver status in dairy cattle by reducing certain enzyme levels [17].

The study intended to measure the impact of two distinct choline supplements on lactation output and milk constituents in pregnant and lactating ewes, as well as their influence on lamb development.

## Materials and Methods

### Ethical approval

Animal handling and procedures were conducted according to Mexican government regulations and standards for the use of experimental animals and the guidelines of the Academic Committee under the State Law on Animal Protection for the State of San Luis Potosí México (ONSSLP, 2014; Approval no. Feb2022/002).

### Study period and location

This study was conducted from April to July 2022 at the Faculty of Agronomy and Veterinary of the San Luis Potosi Autonomous University, located in the ejido Palma de la Cruz, municipality of Soledad de Graciano Sanchez, San Luis Matehuala highway Km 14.5 with coordinates 22°14’06.3” LN and -100°51’52. 6” LO.

### Experimental design

Twenty-four Rambouillet ewes, each weighing 63.7 ± 1.7 kg, divided into eight ewes each for control (no choline treatment), herbal thiocholine (Nuproxa México), and 4 g/ewe/day of RPC (Reashure) treatments, were randomly assigned 30 days before – 30-day postpartum. 30 days before parturition and 30 days during lactation, supplemental choline was added to the mixed feed ration (Table-1) [10]. Each ewe was housed in a 3 × 4 m pen with a feeder and a water bowl. Feed was administered daily at 08:00 h and 15:00 h, and intake was documented. The newborn lambs were weighed every week for average daily gain estimation, while ewes were weighed on giving birth and 35 days later.

**Table-1:** Experimental diet and chemical composition.

Item	g/kg
Sorghum stover	340
Soybean meal	120
Whole corn grain	160
Oat grain	110
Cane molasses	90
Sorghum grain	140
Minerals^a^	40
Chemical composition	
Dry matter (%)	87.66
Crude protein (%)	12.38
Rumen degradable protein (%)^b^	7.06
Neutral detergent fiber (NDF, %)	35.29
Acid detergent fiber (ADF, %)	21.65
ME (Mcal/kg)^b^	2.34

a Mineral: Ca 115 g, P 80 g, Mg 20 g, Na 150 g, Cl 230, K 5 g, S 40 g, Mn 2000 mg, Fe 5500 mg, Zn 6000 mg, Se 30 mg, Co 50 mg, I 100 mg, Cu 1000 mg, Vitamin A 500,000 IU, Vitamin D 150,000 IU and Vitamin E 1000 IU Ca, Vitamin A 500,000 IU, Vitamin D 150,000 IU and Vitamin E 1000 IU.

b Estimated according to the NRC[10]

### Milk analysis

Milk production was assessed every 7 days, according to Reynolds *et al*. [18]. At 0700 h, ewes were milked following an oxytocin injection (20 IU, oxytocin, Aranda®, México) into their jugular veins. The ewe lambs were given this milk. 3 h after the first milking, the ewes were milked again, oxytocin was used, and the yield was recorded. Milk samples were stored at −20°C before further analysis. Samples were analyzed using a Lactoscan Ultrasonic milk analyzer after being mixed, homogenized in a water bath with a temperature set at 40°C for 1 min, and cooled down to 29°C. 2:1 chloroform-methanol (20%) was utilized for lipid extraction from milk before fatty acid (FA) analysis. A 10–20 mg sample of extracted lipid was derivatized using 1:4 (vol vol-1) tetramethylguanidine and methanol [19] after including heptadecanoic acid (17:0) as an internal standard. The FA profiles were analyzed using a gas chromatograph 6890 (Agilent Technologies® United States) equipped with a Supelco-2560, 100 m × 0.25 mm × 0.20 μm column (Sigma Aldrich, Oakville, ON, Canada) and determined by flame ionization detection and splitless injection employing conventional standards (Sigma Aldrich).

### Statistical analysis

All statistical analyses were performed using SAS/STAT software version 9.2 (SAS Institute Inc., Cary, NC) through a MIXED Model procedure. The Tukey test was used to compare the means. p < 0.05 was considered statistically significant.

## Results

Ewes given supplemental choline sources weighed more at lambing (p < 0.05) and their lambs weighed more on 30-day lactation than control ewes. The milk production of ewes given choline from both sources was statistically significantly higher (p < 0.05) compared to the control group (Table-2). Thirty-day offspring growth was significantly greater (p < 0.05) for lambs born to ewes supplemented with choline compared to the control group. Choline supplementation did not alter the main milk components, as shown in Table-2. Choline supplementation brought about alterations in the FA profile (Table-3). Herbal choline decreased the concentrations of short-chain FAs, including caproic, caprylic, capric, lauric, and myristic acids (p < 0.05), while RPC boosted the levels of palmitoleic, heptadecanoic, and heptadecanoic acids. Supplementing with both choline sources raised (p < 0.05) the levels of oleic and cis-11-eicosenoic acid.

**Table-2:** Effect of choline supplementation on ewes and kid performance.

Parameter	Control	RPC	Herbal choline	SEM
BW lambing (kg)	60.3c	63.4^b^	67.4^a^	1.73
BW day 30 (kg)	57.0c	62.0^a^	60.1^a^	1.77
Feed intake (kg/day)	3.09	2.88	2.81	2.71
Milk yield (kg/day)	1.02^b^	1.39^a^	1.57^a^	0.03
Milk composition (g/kg)				
Fat	59.2	58.4	52.4	4.7
Protein	31.1	31.8	28.9	1.2
NFS	84.8	86.4	78.7	3.6
Lactose	46.6	47.5	43.3	1.9
Total solids	221.9	224.2	203.5	7.5
Lambs				
Initial BW kg	4.72^b^	5.7^a^	5.78^a^	0.08
Final BW kg	11.4^b^	13.91^a^	13.58^a^	0.15
Average daily gain g	190	234	222	11.17

RPC=Rumen-protected choline, BW=Body weight, NFS=Non-fat solids, SEM=Standard error of the mean, ^a,b,c^ Means within a row with different superscripts differ (p ≤ 0.05)

**Table-3:** Effects of choline on fatty acid composition of milk fat.

Fatty acid g/100 g	Control	RPC	Herbal choline	SEM
Caproic (C6:0)	2.29^a^	2.11^ab^	1.61^b^	0.14
Caprylic (C8:0)	2.43^a^	2.02^ab^	1.49^b^	0.19
Capric (C10:0)	6.72^a^	5.42^ab^	3.80^b^	0.55
Lauric (C12:0)	3.61^a^	2.87^ab^	2.21^b^	0.24
Myristic (C14:0)	8.85	7.62	7.38	0.46
Pentadecanoic (C15:0)	1.09	1.03	0.78	0.11
Palmitic (C16:0)	24.59	25.14	25.46	0.73
Palmitoleic (C16:1)	1.65^b^	1.86^a^	1.74^ab^	0.05
Heptadecanoic (C17:0)	0.57^b^	0.77^a^	0.70^ab^	0.03
Cis-10-H heptadecanoic (C17:1)	0.57^b^	0.77^a^	0.70^ab^	0.03
Stearic (C18:0)	12.27	12.94	13.41	0.81
Oleic (C18:09c)	29.35^b^	30.67^ab^	34.06^a^	1.14
Linolelaidic (C18:2n6t)	0.28	0.29	0.33	0.04
Linoleic (C18:2n6c)	2.53	2.46	2.65	0.11
Arachidic (C20:0)	0.22	0.29	0.22	0.03
Cis-11-Eicosenoic (C20:1)	0.55^a^	0.71^b^	0.71^b^	0.02
Cis 9 trans 11 CLA	0.45^a^	0.60^b^	0.48^a^	0.05
Arachidonic	0.16	0.33	0.21	0.05

RPC=Rumen-protected choline, BW=Body weight, NFS=Non-fat solids, SEM=Standard error of the mean, ^a,b,c^ Means within a row with different superscripts differ (p ≤ 0.05)

## Discussion

The protein and energy demands, as well as methylation needs, are particularly high during the late stages of sheep pregnancy [9], possibly accounting for the observed weight increase in choline-supplemented sheep [4]. During late pregnancy and early lactation, choline supplementation can heighten methionine accessibility for protein synthesis or economize methionine requirements for choline production [20]. Zhou *et al*. [21] found that choline supplementation did not elevate methionine synthesis.

Providing a pregnant dam with ample choline, methionine, and Vitamin B12 ensures enhanced immunity for their offspring [1]. Newberne [22] discovered that offspring born from dams fed diets deficient in lipotropic compounds weighed less at birth and weaning than those born from rats fed a sufficient diet. In contrast to our findings, Birch *et al*. [23] reported higher birth weights (6.15 kg) for RPC-treated ewes (10 mg choline dosage from trimester 1 to gestation day 133) than the control group (5.16 kg and 5.74 kg for the saline control group). Supriyati *et al*. [15] found no difference in pre-parturition BW of goats given 0, 4, or 8 g/day RPC. Supplementation of choline during late gestation enhances gluconeogenesis, the primary process generating endogenous glucose from substrates, including propionate, lactic acid, and gluconeogenic amino acids, after weaning [24]. Choline supplementation is believed to enhance energy metabolism by increasing the amount of glucose accessible to cells [1, 25]. Leal *et al*. [26] reported that thiocholine supplementation decreases creatine kinase plasma concentration, enabling improved glycolytic control and energy metabolism [27]. Epigenetic mechanisms can respond to this mechanism. 5-hmC levels, reported by Masala *et al*. [28], suggest an active role in demethylation and hydroxymethylation during oocyte growth, alongside stable methylation percentages. Roque-Jiménez *et al*. [29] identified distinct whole-blood 5-hmC percentage changes related to pregnancy and lactation due to bioactive substances in the herbal choline formulas.

In milk production, a positive response to RPC occurs in both dairy cows and goats [17, 29]. Few ruminant studies have utilized a herbal source of RPC [14]. For 90 days following calving, Holstein cows were given 15 g/day of BCho by Mendoza *et al*. [30]. Milk production increased by 3% using the same herbal product and dosage. Ferretiz *et al*. [17] reported that dairy cows produced 9% more fat-corrected milk after 56 days. Herbal compounds, as reported by Chandra *et al*. [31], contain active substances that influence secretory cells in the mammary glands, leading to enhanced milk production and modifications in the composition of non-fat solids and protein. Supplementing BCho during pregnancy enhanced the production and quality of colostrum and milk due to bioactive components in herbal formulas.

Consuming thiocholine results in a higher concentration of propionate in the rumen, which enhances the efficiency of converting dietary feed into energy and improves milk production [26]. The increase in circulating phosphatidylcholine would stimulate the growth of this group of protozoa, consequently decreasing the concentration of ruminal bacteria because protozoa are capable of bacterial engulfment [32].

Choline alters ruminal fermentation [33]. The liver’s effectiveness in metabolizing FAs might be enhanced due to its lipotropic function [34]. Very low-density lipoproteins (VLDLs) produced by the liver are closely associated with the availability of choline and phosphatidylcholine [35]. Phosphatidylcholine enhances hepatic VLDL production, triglyceride availability, and milk yield. The hepatic pathway through which an organism obtains choline is where phosphatidylethanolamine methylation by the enzyme N-methyltransferase occurs, generating phosphatidylcholine that corresponds to approximately 95% of choline-containing compounds in the liver [36].

Choline has little impact on milk’s long-chain FA composition. The medium-chain FA content in milk of unsupplemented sheep is similar to, while higher in goats supplemented with RPC [37]. Certain FAs, including lauric, myristic, and palmitic acids, have been shown to raise low-density lipoprotein (LDL) levels in the blood [38]. The observed decrease from the herbal choline source can be considered beneficial.

Ruminants’ liver FA metabolism influences milk FA profile [39] due to demand for methyl donors (methionine, choline, and betaine) during negative energy balance (fatty liver) [12]. The effect of dietary supplementation with rumen-protected methionine and choline [40] on FA metabolism in the livers of high-performance animals during the peripartum period under negative energy balance is positive. In ewes, goats, and cows, milk FA profiles exhibited insignificant variations when dietarily provided with rumen-protected methyl donors [41–43].

Providing RPC supplementation to pregnant ewes is essential for preventing pregnancy toxemia due to ewe susceptibility and common risk factors, including multiple gestations, low-quality forage, parasites, lack of exercise, and obesity. Michailoff *et al*. [44] demonstrated that RPC supplementation, providing 25 g/day from day 110 of pregnancy until birth, mitigated both negative energy balance impacts and ketosis occurrence in late gestation for dairy sheep.

## Conclusion

Thirty-day post-parturition and initial body weight at lambing were positively influenced by choline supplementation. Supplementing ewes during lactation increased their milk yield, thus benefiting the daily gains of suckling lambs. Choline supplementation alters FA composition. Herbal sources raise short-chain FA levels, while RCP alters medium-chain FA amounts, and both influence long-chain FA content.

## Authors’ Contributions

MMCG: Conceptualization and validation, methodology, investigation, visualization, data analysis, formal analysis, and writing of the original manuscript. GDM: Methodology, sample collection, investigation, visualization, and data analysis. PAHG: Conceptualization and validation, project administration and funding acquisition, supervision, and writing-review and editing. JAMG:Visualization, software and resources, methodology, and writing-review and editing. AVV: Methodology, investigation, and sample collection. ROCL: Methodology, investigation, and sample collection. HALR: Methodology, investigation, sample collection, data analysis, visualization, and resources. All authors have read and agreed to the published version of the manuscript.
